# Supplementary material for: Polarity and mixed-mode oscillations may underlie different patterns of cellular migration
Source: Sci Rep. 2023 Mar 14;13:4223. doi: 10.1038/s41598-023-31042-8 (PMC10014943; doi:10.1038/s41598-023-31042-8)
Supplement: Supplementary file 1 — Supplementary Information. [file 41598_2023_31042_MOESM1_ESM.pdf]

Supplementary Material: Polarity and mixed-mode oscillations may underlie different patterns of cellular migration

Lucie Plazen, Jalal Al Rahbani, Claire M. Brown, Anmar Khadra

S0.1 Derivations of the original six-variable (6V) model

According to the original framework of the 6V model [1,2], Rac and Rho exist in the form of either active/membrane-bound state (i.e., RacGTP and RhoGTP) and inactive/cytosolic state (i.e., RacGDP and RhoGDP). It is hypothesized that the active forms of these proteins mutually inhibit each other's activation [3]; this latter feature represents the key element of the original 6V model.

The connection between paxillin S273 phosphorylation and elevated Rac activity is both active PAK (i.e., RacGTP-bound PAK) and PIX. Active PAK phosphorylates paxillin, whereas PIX is a known RacGEF that is bound to the adhesion as part of the GIT-PIX-PAK complex. As the level of S273-phosphorylated paxillin (Pax_p) increases, so does the binding of PIX to the adhesion. The increased GEF activity that results from PIX accumulation causes a rise in the Rac activation rate.

To derive the equations that describe the dynamics of this system, we will follow the same steps outlined in [2]. These equations are based on a set of reactions that are all listed in [2]

Crosstalk between the two proteins Rac and Rho (i.e., their mutual inhibition of each other) is modelled as a cooperative reduction of their activation rate. In the case of Rac, its RacGEF-dependent activation is expressed in terms of a decreasing Hill function that depends on the concentration of active Rho ($[RhoGTP]$), with a Hill coefficient n and a half-maximal inhibition L_{Rho}, whereas Rac inactivation follows mass-action kinetics and is dependent on RacGAP (assumed to be constant). Based on this, the equations governing the dynamics of active and inactive Rac concentrations ($[RacGTP]$ and $[RacGDP]$, respectively) are given by

$$\begin{aligned}
\frac{[RacGTP]}{dt} &= k^+_{Rac}[RacGEF]\left(1 - \frac{[RhoGTP]^n}{L^n_{Rho} + [RhoGTP]^n}\right)[RacGDP] \\
&\quad -k^-_{Rac}[RacGAP][RacGTP] + k^-_{PAK}\Big([PAK - RacGTP] + [Pax_p - GIT - PIX - PAK - RacGTP]\Big) \\
&\quad -k^+_{PAK}\Big([PAK] + [Pax_p - GIT - PIX - PAK]\Big)[RacGTP] \\
\frac{[RacGDP]}{dt} &= -k^+_{Rac}[RacGEF]\left(1 - \frac{[RhoGTP]^n}{L^n_{Rho} + [RhoGTP]^n})\right)[RacGDP] + k^-_{Rac}[RacGAP][RacGTP]
\end{aligned}$$

The dynamics of active and inactive Rho concentrations ($[RhoGTP]$ and $[RhoGDP]$, respectively) are modelled similarly. In this case, the RhoGEF-dependent Rho activation is expressed in terms of a decreasing Hill function that depends on the concentration of active Rac ($[RacGTP]$), with a Hill coefficient n and a half-maximal inhibition L_{Rac}, and its inactivation follows mass-action kinetics that is dependent on RhoGAP (assumed to be

constant). With this in mind, we obtain the following equations

$$\begin{aligned}
\frac{[RhoGTP]}{dt} &= k_{Rho}^{+}[RhoGEF] \\
&\times\left(1-\frac{\Big([RacGTP]+[PAK-RacGTP]+[Pax_p-GIT-PIX-PAK-RacGTP]\Big)^n}{L_{Rac}^n+\Big([RacGTP]+[PAK-RacGTP]+[Pax_p-GIT-PIX-PAK-RacGTP]\Big)^n}\right) \\
&\times[RhoGDP]-k_{Rho}^{-}[RhoGAP][RhoGTP] \\
\frac{[RhoGDP]}{dt} &= -k_{Rho}^{+}[RhoGEF] \\
&\times\left(1-\frac{\Big([RacGTP]+[PAK-RacGTP]+[Pax_p-GIT-PIX-PAK-RacGTP]\Big)^n}{L_{Rac}^n+\Big([RacGTP]+[PAK-RacGTP]+[Pax_p-GIT-PIX-PAK-RacGTP]\Big)^n}\right) \\
&\times[RhoGDP]+k_{Rho}^{-}[RhoGAP][RhoGTP].
\end{aligned}$$

It is important to note that, since PIX is a RacGEF, the total RacGEF concentration ($[RacGEF]$) is given by

$$\begin{aligned}
[RacGEF] &= [RacGEF_b]+[PIX]+[GIT-PIX]+[PIX-PAK]+[GIT-PIX-PAK] \\
&+[Pax_p-GIT-PIX-PAK]+[Pax_p-GIT-PIX-PAK-RacGTP],
\end{aligned}$$

where $[RacGEF_b]$ is the concentration of basal level of RacGEFs (assumed to be constant).

The reactions governing the dynamics of paxillin include paxillin phosphorylation/dephosphorylation, and binding/unbinding to/from the GIT-PIX-PAK complex. The phosphorylation of this protein is driven by active PAK (i.e., RacGTP-bound PAK) and is expressed as a Hill function with a Hill coefficient n and half-maximum activation L_{PAK}, whereas its dephosphorylation follows mass-action kinetics and depends on the concentration of the phosphatase PP2A (assumed to be constant). By letting $[Pax_p]$ and $[Pax]$ denote the concentrations of phosphorylated and unphosphorylated paxillin, respectively, we obtain the following equations for these two molecular species

$$\begin{aligned}
\frac{[Pax_p]}{dt} &= B\frac{\Big([PAK-RacGTP]+[Pax_p-GIT-PIX-PAK-RacGTP]\Big)^n}{L_{PAK}^n+\Big([PAK-RacGTP]+[Pax_p-GIT-PIX-PAK-RacGTP]\Big)^n}[Pax] \\
&-k_{Pax}^{-}[PP2A][Pax_p]+k_C^{-}[Pax_p-GIT-PIX-PAK] \\
&-k_C^{+}[Pax_p][GIT-PIX-PAK] \\
\frac{[Pax]}{dt} &= -B\frac{\Big([PAK-RacGTP]+[Pax_p-GIT-PIX-PAK-RacGTP]\Big)^n}{L_{PAK}^n+\Big([PAK-RacGTP]+[Pax_p-GIT-PIX-PAK-RacGTP]\Big)^n}[Pax] \\
&+k_{Pax}^{-}[PP2A][Pax_p],
\end{aligned}$$

where B is paxillin maximum phosphorylation rate.

The equations governing the dynamics of all intermediates considered in this system are listed below (see [2]

for the whole list of chemical reactions that produce these equations)

$$\frac{d[PAK]}{dt} = k^-_{PAK}[PAK - RacGTP] - k^+_{PAK}[PAK][RacGTP] + k^-_X[GIT - PIX - PAK]$$
$$- k^+_X[GIT - PIX][PAK] + k^-_X[PIX - PAK] - k^+_X[PIX][PAK]$$

$$\frac{d[PAK - RacGTP]}{dt} = -k^-_{PAK}[PAK - RacGTP] + k^+_{PAK}[PAK][RacGTP] + k^-_X[PIX - PAK - RacGTP]$$
$$- k^+_X[PIX][PAK - RacGTP]$$

$$\frac{d[GIT - PIX]}{dt} = k^+_G[GIT][PIX] - k^-_G[GIT - PIX] + k^-_X[GIT - PIX - PAK] - k^+_X[GIT - PIX][PAK]$$

$$\frac{d[PIX - PAK]}{dt} = k^+_X[PIX][PAK] - k^-_X[PIX - PAK] + k^-_G[GIT - PIX - PAK] - k^+_G[GIT][PIX - PAK]$$
$$+ k^-_{PAK}[PIX - PAK - RacGTP] - k^+_{PAK}[PIX - PAK][RacGTP]$$

$$\frac{d[PIX - PAK - RacGTP]}{dt} = k^+_X[PIX][PAK - RacGTP] - k^-_X[PIX - PAK - RacGTP]$$
$$+ k^+_{PAK}[PIX - PAK][RacGTP] - k^-_{PAK}[PIX - PAK - RacGTP]$$

$$\frac{d[GIT - PIX - PAK]}{dt} = k^+_X[GIT - PIX][PAK] - k^-_X[GIT - PIX - PAK] + k^+_G[GIT][PIX - PAK]$$
$$- +k^-_G[GIT - PIX - PAK] + k^-_C[Pax_p - GIT - PIX - PAK]$$
$$- k^+_C[Pax_p][GIT - PIX - PAK]$$

$$\frac{d[Pax_p - GIT - PIX - PAK]}{dt} = k^+_C[Pax_p][GIT - PIX - PAK] - k^-_C[Pax_p - GIT - PIX\text{-}PAK]$$
$$+ k^-_{PAK}[Pax_p - GIT - PIX - PAK - RacGTP]$$
$$- k^+_{PAK}[Pax_p - GIT - PIX - PAK][RacGTP]$$

$$\frac{d[Pax_p - GIT - PIX - PAK - RacGTP]}{dt} = -k^-_{PAK}[Pax_p - GIT - PIX - PAK - RacGTP]+$$
$$k^+_{PAK}[Pax_p - GIT - PIX - PAK][RacGTP].$$

After setting these latter equations to steady state using quasi-steady-state approximation, the concentrations of each intermediate can be expressed as a function of $[RacGTP]$, $[Pax_p]$, $[GIT]$, $[PIX]$ and $[PAK]$, where $[GIT]$

and $[PIX]$ are assumed to be constant. These steady state expressions are given by

$$
\begin{aligned}
&[PAK-RacGTP] = \frac{k^+_{PAK}}{k^-_{PAK}}[PAK][RacGTP]\\
&[GIT-PIX] = \frac{k^+_G}{k^-_G}[GIT][PIX] = constant\\
&[PIX-PAK] = \frac{k^+_X}{k^-_X}[PIK][PAK]\\
&[PIX-PAK-RacGTP] = \frac{k^+_X}{k^-_X}\frac{k^+_{PAK}}{k^-_{PAK}}[PIK][PAK][RacGTP]\\
&[Pax_p-GIT-PIX-PAK] = \frac{k^+_G}{k^-_G}\frac{k^+_X}{k^-_X}\frac{k^+_C}{k^-_C}[GIT][PIK][PAK][Pax_p]\\
&[Pax_p-GIT-PIX-PAK-RacGTP] = \frac{k^+_G}{k^-_G}\frac{k^+_X}{k^-_X}\frac{k^+_C}{k^-_C}\frac{k^+_{PAK}}{k^-_{PAK}}[GIT][PIK][PAK][Pax_p][RacGTP].
\end{aligned}
$$

The expression for the steady state of $[PAK]$ is derived from the total concentration of PAK (PAK_{tot}; assumed to be constant), as follows

$$
\begin{aligned}
[PAK_{tot}] = {} & [PAK] + [PIX-PAK] + [GIT-PIX-PAK] + [Pax_p-GIT-PIX-PAK] + [PAK-RacGTP]\\
& + [PIX-PAK-RacGTP] + [Pax_p-GIT-PIX-PAK-RacGTP].
\end{aligned}
$$

Substituting the steady state concentrations of the various intermediates appearing in the latter equation, we obtain

$$
\begin{aligned}
\frac{[PAK_{tot}]}{[PAK]} = {} & k_X[PIX] + k_Gk_X[GIT][PIX] + k_Gk_Xk_C[GIT][PIX][Pax_p] + \alpha k_X[PIX][RacGTP]\\
& + \alpha k_Gk_Xk_C[GIT][PIX][Pax_p][RacGTP] + 1 + \alpha[RacGTP],
\end{aligned}
$$

where $k_G = \frac{k^+_G}{k^-_G}$, $k_X = \frac{k^+_X}{k^-_X}$, $k_C = \frac{k^+_C}{k^-_C}$ and $\alpha = \frac{k^+_{PAK}}{k^-_{PAK}}$. These newly defined parameters represent the association constant for $PIX-PAK$ binding (k_X), the association constant for $GIT-PIX$ binding (k_G), the association constant for Pax_p-GIT binding (k_C) and the affinity constant for $PAK-RacGTP$ binding (α_R). Solving for $[PAK]$ and rearranging, we obtain

$$
[PAK] = \frac{[PAK_{tot}]}{(1 + k_X[PIX] + k_Gk_Xk_C[GIT][PIX][Pax_p])(1+\alpha[RacGTP]) + k_Gk_X[GIT][PIX]}
$$

This latter expression for $[PAK]$ can be used to derive the ratio of $[PAK]$-to-$[PAK_{tot}]$ (denoted by K^*_i) as follows

$$
K^*_i = \frac{[PAK]}{[PAK_{tot}]} = \{(1 + k_X[PIX] + k_Gk_Xk_C[GIT][PIX][Pax_p])(1+\alpha[RacGTP]) + k_Gk_X[GIT][PIX]\}^{-1}.
$$

It follows that the scaled concentration of active PAK (donated by $[PAK^*]$) is given by

$$
K = \frac{[PAK^*]}{[PAK]_{tot}} = \alpha_R R\left(1 + k_x[PIX] + k_Gk_Xk_C[GIT][PIX][Pax_{tot}]P)\right) K^*_i.
$$

Since PIX is a known RacGEF, bound to the adhesion as part of the GIT-PIX-PAK complex, we can define PIX-dependent Rac activation I^*_K as follows

$$
\begin{aligned}
I^*_K = {} & I'_K([PIX-PAK] + [GIT-PIX-PAK] + [Pax_p-GIT-PIX-PAK] + [PIX-PAK-RacGTP]\\
& + [Pax_p-GIT-PIX-PAK-RacGTP]),
\end{aligned}
$$

where I'_K is the PIX-mediated rate of Rac activation. Substituting the concentrations of each intermediate species using their concentrations at steady state, we obtain

$$
\begin{aligned}
I^*_K = {} & I'_K([PAK](k_X[PIX] + k_Gk_X[GIT][PIX] + k_Gk_Xk_C[GIT][PIX][Pax_p] + \alpha k_X[PIX][RacGTP]\\
& + \alpha k_Gk_Xk_C[GIT][PIX][Pax_p][RacGTP] + 1 + \alpha[RacGTP]))\\
& + I'_K([PAK](\frac{[PAK_{tot}]}{[PAK]} - 1 - \alpha[RacGTP])) + I'_K[PAK_{tot}](1\frac{[PAK]}{[PAK_{tot}]} - \alpha\frac{[PAK][RacGTP]}{[PAK_{tot}]}).
\end{aligned}
$$

Letting $I_K = I'_K[PAK_{tot}]$, $R = \frac{[RacGTP]}{[Rac_{tot}]}$ and $\alpha_R = \alpha[Rac_{tot}]$, where $[Rac_{tot}]$ is the total concentration of Rac (assumed constant), we get

$$I_K^* = I_K(1 - K_i^*(1 + \alpha_R R)).$$

To determine the expression level of unphosphorylated paxillin, P_i, we first assume a constant total concentration of paxillin ($[Pax_{tot}]$), which includes unphosphorylated and phosphorylated paxillin and any complexes which contain them. Given this assumption, the concentration of unphosphorylated paxillin is

$$[Pax] = [Pax_{tot}] - [Pax_p] - [Pax_p - GIT - PIX - PAK] - [Pax_p - GIT - PIX - PAK - RacGTP]$$

After substituting the steady state expressions for the intermediate complexes, we obtain

$$[Pax] = [Pax_{tot}] - [Pax_p](1 + k_G k_X k_C [GIT][PIX][PAK](1 + \alpha[RacGTP])).$$

Scaling the latter equation by $[Pax_{tot}]$ and substituting the scaled variables $P = \frac{[Pax_p]}{[Pax_{tot}]}$, $P_i = \frac{[Pax]}{[Pax_{tot}]}$ give

$$P_i = 1 - P(1 + k_G k_X k_C [GIT][PIX][PAK_{tot}]K_i^*(1 + \alpha_R R).$$

Finally, scaling $[RhoGTP]$ and $[RhoGDP]$ by total Rho concentration ($[Rho_{tot}]$ (assumed constant) and letting $\rho = \frac{[RhoGTP]}{[Rho_{tot}]}$ and $\rho_i = \frac{[RhoGDP]}{[Rho_{tot}]}$, as well as scaling $[RacGDP]$ by $[Rac_{tot}]$ and letting $R_i = \frac{[RacGDP]}{[Rac_{tot}]}$, we obtain a scaled 6V model whose dynamics in the presence of diffusion is given by

$$\begin{aligned}
\frac{\partial R}{\partial t} &= (I_R + I_K^*)\left(\frac{L_\rho^n}{L_\rho^n + \rho^n}\right) R_i - \delta_R R + D_R \frac{\partial^2 R}{\partial x^2} \\
\frac{\partial R_i}{\partial t} &= -(I_R + I_K^*)\left(\frac{L_\rho^n}{L_\rho^n + \rho^n}\right) R_i + \delta_R R + D_{R_i} \frac{\partial^2 R_i}{\partial x^2} \\
\frac{\partial \rho}{\partial t} &= I_\rho \left(\frac{L_R^n}{L_R^n + (R + \gamma K)^n}\right) \rho_i - \delta_\rho \rho + D_\rho \frac{\partial^2 \rho}{\partial x^2} \\
\frac{\partial \rho_i}{\partial t} &= -I_\rho \left(\frac{L_R^n}{L_R^n + (R + \gamma K)^n}\right) \rho_i + \delta_\rho \rho + D_{\rho_i} \frac{\partial^2 \rho_i}{\partial x^2} \\
\frac{\partial P}{\partial t} &= B\left(\frac{K^n}{L_K^n + K^n}\right) P_i - \delta_P P + D_P \frac{\partial^2 P}{\partial x^2} \\
\frac{\partial P_i}{\partial t} &= -B\left(\frac{K^n}{L_K^n + K^n}\right) P_i + \delta_P P + D_{P_i} \frac{\partial^2 P_i}{\partial x^2},
\end{aligned}$$

where D_x ($x = R, R_i, \rho, \rho_i, P, P_i$) is the diffusion coefficient of each molecular species and $\gamma = \frac{[PAK_{tot}]}{[Rac_{tot}]}$.

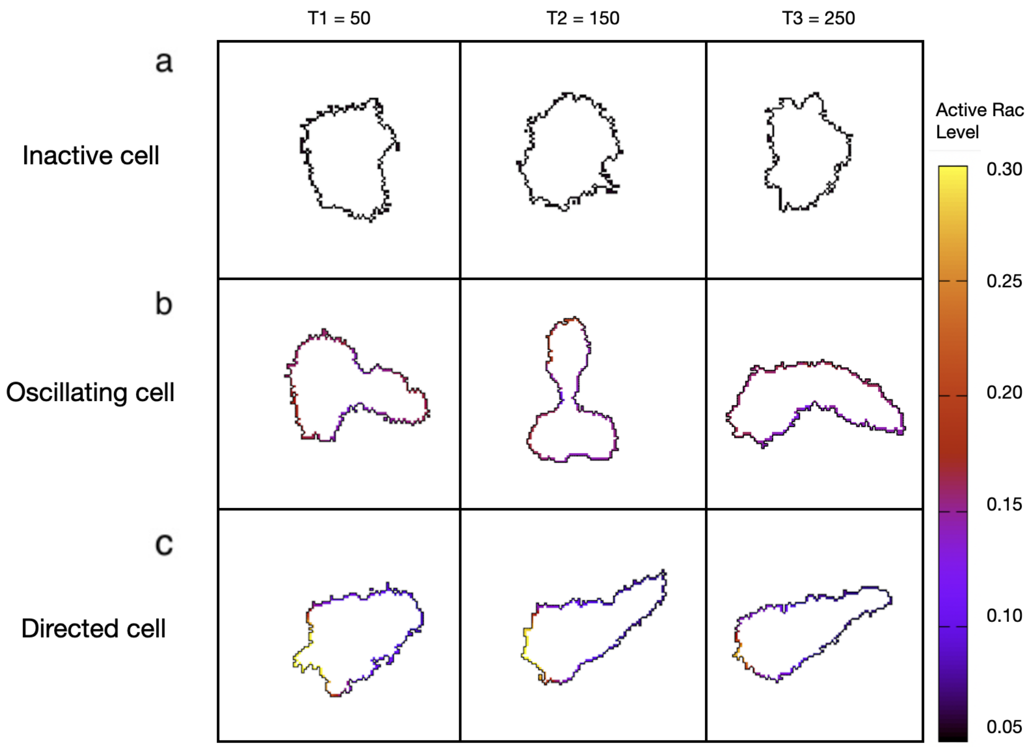

Figure S1: Membrane bound scaled active Rac concentration in simulated CPM cells defines the shapes of (a) inactive, (b) oscillating, and (c) directed cells. Simulations are shown at three different time points: $T_1 = 50$, $T_2 = 150$ and $T_3 = 250$ time-steps. Scaled active Rac concentration is colour-coded according to the colour-bar to the right.

Parameter	Description	Value	Unit	References
I_R	Basal Rac activation Rate	0.0035	s^{-1}	[1]
δ_R	Rac inactivation rate	0.025	s^{-1}	[1]
L_ρ	Rho-dependent half-maximum inhibition of Rac	0.34	unitless	[1]
I_ρ	Basal Rho activation Rate	0.016	s^{-1}	[1]
δ_ρ	Rho inactivation rate	0.016	s^{-1}	[1]
L_R	Rac-dependent half-maximum inhibition of Rho	0.34	unitless	[1]
γ	Ratio of total PAK to total Rac	0.3	unitless	[1]
δ_P	Paxillin dephosphorylation rate	0.00041	s^{-1}	[1]
n	Hill coefficient	4	unitless	[1]
α_P	Linearization coefficient in paxillin activation	2.7	unitless	[1]
L_K	Active PAK-dependent half-maximum activation of paxillin	5.77	unitless	[1]
I_K	Additional Rac activation due to paxillin	0.009	s^{-1}	[1]
k_G	Association constant for GIT-PIX binding	5.71	s^{-1}	[1]
$[GIT]$	Concentration of GIT	0.11	μM	[1]
k_X	Association constant for PIX-PAK binding	41.7	s^{-1}	[1]
$[PIX]$	Concentration of PIX	0.069	μM	[1]
k_C	Association constant for Pax_p-GIT binding	5	s^{-1}	[1]
$[Pax_{tot}]$	Total concentration of paxillin	2.3	μM	[1]
α_R	Affinity constant for PAK-RacGTP binding	15	unitless	[1]
D_R	Diffusion coefficient of active Rac	0.0025	$\mu m^2.s^{-1}$	[1]
D_{R_i}	Diffusion coefficient of inactive Rac	0.43	$\mu m^2.s^{-1}$	[1]
ϵ	Time constant for B	0.01	s^{-1}	Estimated
B_r	Resting state of B	10	unitless	Estimated
k_B	Recovery rate of B back to its resting state	0.04	unitless	Estimated
γ_R	Strength of R feedback onto B	8.6956	s^{-1}	Estimated
η, ϵ_B	Parameters that guarantee the positivity of B	10^4, 10^{-4}	unitless	Estimated
ϵ_L	Time constant of k_B	10^{-5}	s^{-1}	Estimated
γ_K	Source term	0.15	unitless	Estimated

Table S1: Summary of parameter values.

Parameter	Description	Value	References
P_{target}	Targeted perimeter	200	Estimated, [4]
λ_P	Weights describing membrane deformation resistance	0.002	Estimated and fitted
S_{target}	Targeted surface	1200	Estimated, [5]
λ_S	Weights describing membrane deformation resistance	0.0004	Estimated and fitted
λ_{Act}	Weights describing protrusive strength	2	Estimated and fitted
MCS Duration	Ratio between Monte Carlo Step and simulation time	0.5	
Temperature	Boltzmann probability to accept updates that increase energy	0.12	Fitted
Yield	Offset for Boltzmann probability distribution representing resistance to membrane deformations	0.1	Fitted, [6]

Table S2: Summary of CPM parameter values.

References

[1] Tang, K., Boudreau, C. G., Brown, C. M. & Khadra, A. Paxillin phosphorylation at serine 273 and its effects on rac, rho and adhesion dynamics. *PLoS computational biology* **14**, e1006303 (2018).

[2] Tang, K. *Mathematical model of paxillin phophorylation at serine 273 and its effect on Rac, Rho, and adhesion dynamics* (McGill University (Canada), 2018).

[3] Byrne, K. M. *et al.* Bistability in the rac1, pak, and rhoa signaling network drives actin cytoskeleton dynamics and cell motility switches. *Cell systems* **2**, 38–48 (2016).

[4] Ouchi, N. B., Glazier, J. A., Rieu, J.-P., Upadhyaya, A. & Sawada, Y. Improving the realism of the cellular potts model in simulations of biological cells. *Physica A: Statistical Mechanics and its Applications* **329**, 451–458 (2003).

[5] Graner, F. & Glazier, J. A. Simulation of biological cell sorting using a two-dimensional extended potts model. *Physical review letters* **69**, 2013 (1992).

[6] Käfer, J., Hogeweg, P. & Marée, A. F. M. Moving forward moving backward: directional sorting of chemotactic cells due to size and adhesion differences. *PLoS computational biology* **2**, e56 (2006).
